# Biophysical Characterization of a Novel Phosphopentomutase from the Hyperthermophilic Archaeon *Thermococcus kodakarensis*

**DOI:** 10.3390/ijms252312893

**Published:** 2024-11-30

**Authors:** Zahra Naz, Jacek Lubkowski, Muhammad Saleem, Mehwish Aslam, Moazur Rahman, Alexander Wlodawer, Naeem Rashid

**Affiliations:** 1 School of Biological Sciences, University of the Punjab, Lahore 54590, Pakistan; zahranaz55@ymail.com (Z.N.); msaleem.sbs@pu.edu.pk (M.S.); maslam.sbs@pu.edu.pk (M.A.); moaz.sbs@pu.edu.pk (M.R.); naeem.ff.sbs@pu.edu.pk (N.R.); 2 Center for Structural Biology, National Cancer Institute, National Institutes of Health, Frederic, MD 21702, USA; lubkowsj@mail.nih.gov

**Keywords:** *Thermococcus kodakarensis*, modified pentose phosphate pathway, phosphopentomutase, X-ray crystallography

## Abstract

Phosphopentomutases catalyze the isomerization of ribose 1-phosphate and ribose 5-phosphate. *Thermococcus kodakarensis*, a hyperthermophilic archaeon, harbors a novel enzyme (PPM*_Tk_*) that exhibits high homology with phosphohexomutases but has no significant phosphohexomutase activity. Instead, PPM*_Tk_* catalyzes the interconversion of ribose 1-phosphate and ribose 5-phosphate. Here, we report biophysical analysis, crystallization, and three-dimensional structure determination of PPM*_Tk_* by X-ray diffraction at 2.39 Å resolution. The solved structure revealed a novel catalytic motif, unique to PPM*_Tk_*, which makes this enzyme distinct from the homologous counterparts. We postulate that this novel catalytic motif may enable PPM*_Tk_* to isomerize phosphopentose instead of phosphohexose. To the best of our knowledge, this is the first biophysical and structural analysis of a phosphopentomutase from hyperthermophilic archaea.

## 1. Introduction

The pentose phosphate pathway (PPP) has been known to play a pivotal role in microorganisms that utilize various substrates, including nucleosides/nucleotides or their derivatives, as carbon and energy sources [1]. PPP has been found to connect cellular metabolic pathways. Glucose 6-phosphate, a metabolite of glycolysis, is converted to deoxyribose 5-phosphate (dR5P) in a series of reactions involving glucose-6-phosphate isomerase (GPI, EC:5.3.1.9), phosphofructokinase (PFP, EC:2.7.1.90), fructose-bisphosphate aldolase (FBPA, EC:4.1.2.13), and deoxyribose-phosphate aldolase (DERA, EC:4.1.2.4). 

**Figure 1:**
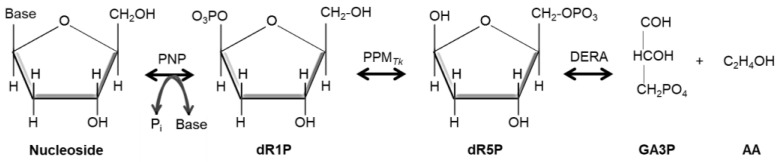
Schematic representation of nucleoside metabolism in *T. kodakarensis*. Symbols: dR1P, deoxyribose 1-phosphate; dR5P, deoxyribose 5-phosphate; GA3P, glyceraldehyde 3-phosphate; AA, acetaldehyde; PNP, purine nucleoside phosphorylase; PPM*_Tk_*, phosphopentomutase from *T. kodakarensis*; DERA, deoxyribose-phosphate aldolase.

Phosphopentomutase (PPM, EC:5.4.2.7) catalyzes the reversible isomerization of dR5P to deoxyribose 1-phosphate (dR1P), which enters in nucleotide metabolism [2] (Figure 1).

Most of the archaea lack typical PPP; instead, they generate dR5P through a modified pentose phosphate pathway [3,4]. A unique PPM (PPM*_Tk_*) has been characterized from the hyperthermophilic archaeon *Thermococcus kodakarensis* [5]. This novel enzyme possesses a distinct primary structure. The open reading frame encoding PPM*_Tk_* lies within COG1109 (the genes encoding phosphohexomutases) instead of the usual COG1015 (the genes encoding phosphopentomutases). It contains the active site motif, “TXSHNP” (serine as the active site residue), and “DXDXDR” (the metal-binding site) conserved in various phosphohexomutases [6]. However, PPM*_Tk_* displayed no significant mutase activity against various hexose substrates including glucose 1-phosphate (G1P), mannose 1-phosphate (M1P), fructose 1-phosphate (F1P), and glucosamine 1-phosphate (Gn1P). In contrast, high activity was observed against a pentose substrate, dR1P (210 μmol min^−1^ mg^−1^). Thermostability analysis has revealed that PPM*_Tk_* is highly stable at higher temperatures with a half-life of 90 min at 100 °C [5].

Among the twenty-three PPMs of bacterial origin, whose 3D structures are determined, 22 are monomeric [7,8]. In contrast, PPM*_Tk_* forms tetramers in solution as indicated by gel filtration analysis [5]. Similarly, the primary structure of PPM*_Tk_* displays no significant similarity to any PPM whose 3D structure has been determined. Furthermore, no 3D structure of a PPM of archaeal origin has been reported to date. Therefore, there is a need for the 3D structure of this novel enzyme. The current study aimed at the crystallization and determination of the 3D structure of PPM*_Tk_*. Here, we report the crystal structure analysis of PPM*_Tk_*, which has been performed at a resolution of 2.39 Å.

## 2. Results

### 2.1. Domain Structure Analysis of PPM_Tk_

Domain structure analysis indicated that a protomer of PPM*_Tk_* was composed of two delineated domains, the larger N-terminal domain and the smaller C-terminal domain. However, the InterProScan [9] and CATH [10] analyses indicated the presence of four domains that corresponded to their counterparts found in hexomutases, particularly in the α-D-phosphohexomutase superfamily (IPR005841) and archaeal-type phosphoglucosamine mutase family (IPR024086). Although the three “domains” present in the larger N-terminal domain of PPM*_Tk_* should properly be called “subdomains”, we will use the term “domain” for the sake of agreement with the previous designations. Based on the description of the domain structure in α-D-phosphohexomutase, the domains in PPM*_Tk_* can be labeled as I (residues 2–129), II (148–244), III (248–353), and IV (360–435). Domains I, II, and III consist of a three-layer α/β/α sandwich each, while domain IV has a two-layer α/β sandwich topology. In hexomutases, domains I and IV are reported to be involved in the intramolecular phosphotransferase activity [11,12], while domain II in the metal binding [6].

### 2.2. Vector Construction, Overexpression, and Purification of PPM_Tk_

PCR, using oligonucleotide primers given in Section 2.3, resulted in the amplification of ~6.9 kbp DNA fragment (Figure S1), matching the size of pET-His_6_-TEV-Tk1777. The amplified gene was treated with *Dpn*I to digest the methylated DNA used as a template for gene amplification. After digestion, the newly synthesized DNA containing the His_6_-tag was used to transform *E. coli* DH5α cells. Plasmid DNA was isolated from one of the transformants and cloning was confirmed by restriction digestion with *Nde*I and *Eco*RI (Figure S2). The sequence of the cloned gene was confirmed by DNA sequencing (Figure S3) and it matched 100% with the desired sequence.

**Figure 2:**
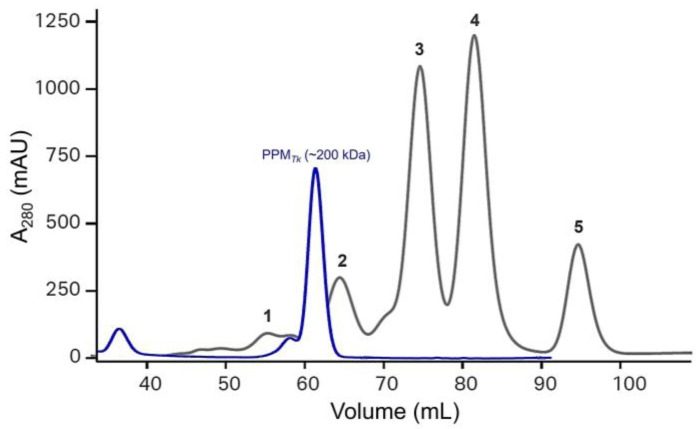
The chromatogram of elution profile of PPM*_Tk_* from gel filtration. Peaks in grey correspond to the standards (1. Ferritin—440 kDa; 2. IgG—158 kDa; 3. Human albumin—66 kDa; 4. Ovalbumin—44 kDa; and 5. Myoglobulin—17 kDa), while the peak in blue is for PPM*_Tk_*.

**Figure 3:**
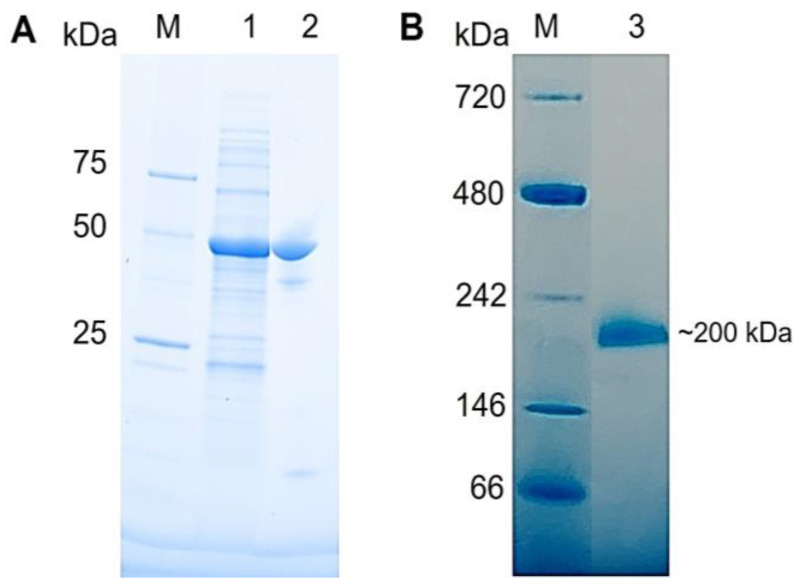
(**A**) SDS-PAGE (**A**), and Native PAGE (**B**) analyses of PPM*_Tk_*. Lane 1, soluble fractions after cell lysis; Lane 2, purified protein after gel-filtration column; Lane 3, purified protein run on Native PAGE; Lane M, protein marker (Bio-Rad, Hercules, CA, USA:_Precision Plus Protein™ Unstained Protein Standards, Cat. #1610363 for SDS-PAGE and NativeMark™ Unstained Protein Standard, Cat. No. LC0725 for Native PAGE).

For recombinant protein production, *E. coli* BL21-CodonPlus^®^ (DE3)-RIPL and Rosetta^TM^ 2(DE3)pLysS Singles^TM^ cells were independently transformed using pET-His_6_-TEV-Tk1777. Induction of heterologous gene expression with 1 mM IPTG (final concentration) resulted in the production of the recombinant PPM*_Tk_* in comparatively high amounts in BL21-CodonPlus^®^ (DE3)-RIPL cells (Figure S4). PPM*_Tk_* was eluted at a retention volume of 61 mL, equivalent to ~200 kDa (Figure 2), when passed through HiLoad^TM^ 16/60 Superdex^TM^ 200 Prep Grade column (Cytiva^TM^), indicating that PPM*_Tk_* is a tetrameric protein. After Ni-NTA and size exclusion chromatography, nearly a homogeneous protein band, equivalent to the calculated molecular weight of PPM*_Tk_*, was observed on SDS-PAGE (Figure 3). Protein purified by size exclusion chromatography was used for further analysis and structural studies.

### 2.3. Electrospray Ionization Mass Spectrometric Analysis of PPM_Tk_

**Figure 4:**
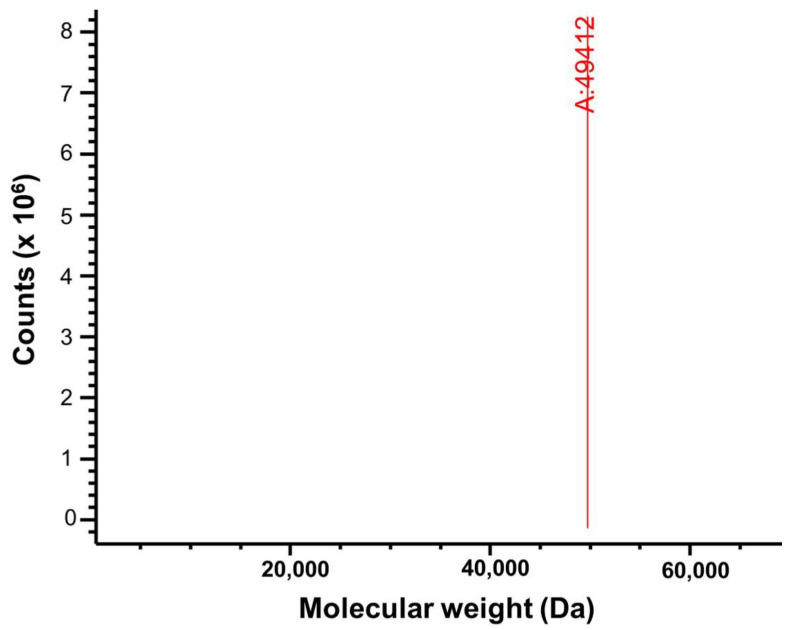
LC-MS analysis of purified PPM*_Tk_*. Mass spectrometry analysis of PPM*_Tk_* was performed after removal of His_6_-tag.

Electrospray ionization mass spectrometry (ESI-MS) generates multiple charged species of a protein which may fragment when activated in the gas phase. Detecting these fragments helps to confirm the purity and molecular weight of the sample protein [13]. When purified PPM*_Tk_* was subjected to electrospray ionization LC-MS analysis, a single peak appeared at a molecular weight of ~49 kDa (Figure 4), matching the subunit molecular weight of PPM*_Tk_*. These results demonstrated the purity and integrity of PPM*_Tk_*.

### 2.4. Mass Photometric Studies of PPM_Tk_

**Figure 5:**
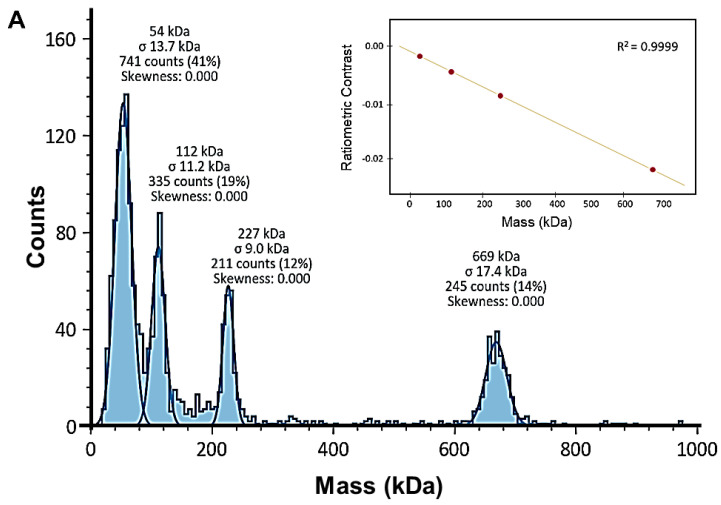

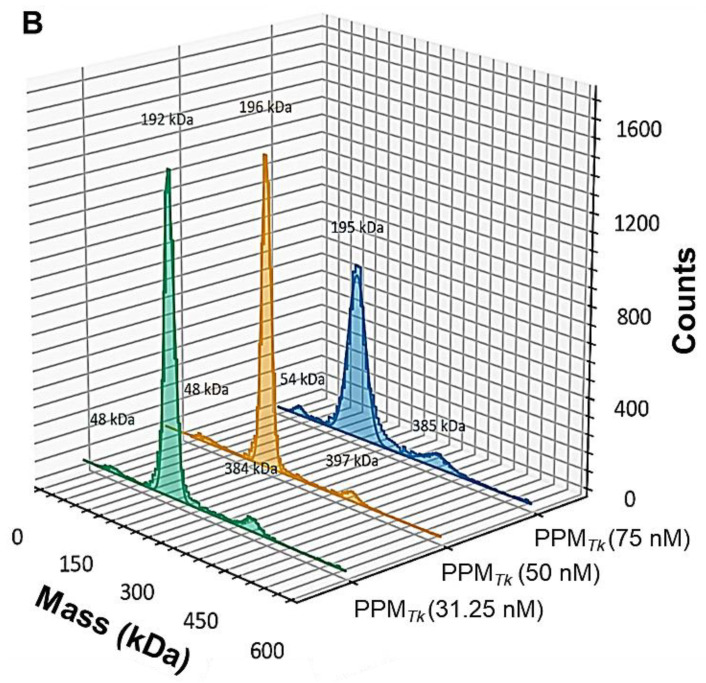
(**A**) Mass photometry analysis of PPM*_Tk_*. (**A**) Histograms of the MP signal distribution. The molecular weights of all peaks were calibrated against the proteins with well-defined molecular weights; multimeric thyroglobulin: 330 kDa, and 630 kDa, and β-amylase: 56 kDa, 112 kDa, and 224 kDa, with an error margin of 1.6%. Inset: linear fit of the contrast-to-mass calibration. (**B**) Histograms of the masses associated with single-molecule surface absorption events for selected protein standards. Histograms represent the masses for PPM*_Tk_* at different concentrations.

Mass photometry was performed to investigate the oligomeric states of PPM*_Tk_* in solution. Data obtained from mass photometric analysis demonstrated the presence of negligible amounts of transient species (monomer and octamer). The majority of the molecules were in tetrameric form (Figure 5) indicating that the active form of PPM*_Tk_* is a tetramer, thus supporting the results of gel filtration chromatography.

### 2.5. Differential Scanning Fluorimetry Analysis of PPM_Tk_

**Figure 6:**
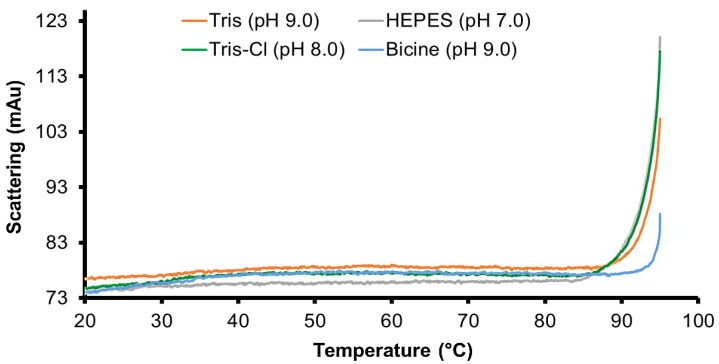
Thermal stability analysis of PPM*_Tk_* in various buffers of different pH using differential scanning fluorimetry.

Differential scanning *fluorimetry* was performed to investigate the thermal and conformational stability of PPM_Tk_ at various pH. The results illustrated that PPM*_Tk_* was stable until 90 °C at all the pH examined. However, it displayed relatively higher stability at pH 9.0. At this pH, PPM*_Tk_* showed higher stability in Bicine buffer, compared to Tris-HCl buffer. It displayed a melting temperature of 95 °C, at pH 9.0, compared to 85 °C, at pH 7–8 (Figure 6).

### 2.6. Secondary Structure Analysis of PPM_Tk_ Using CD Spectrometry

**Figure 7:**
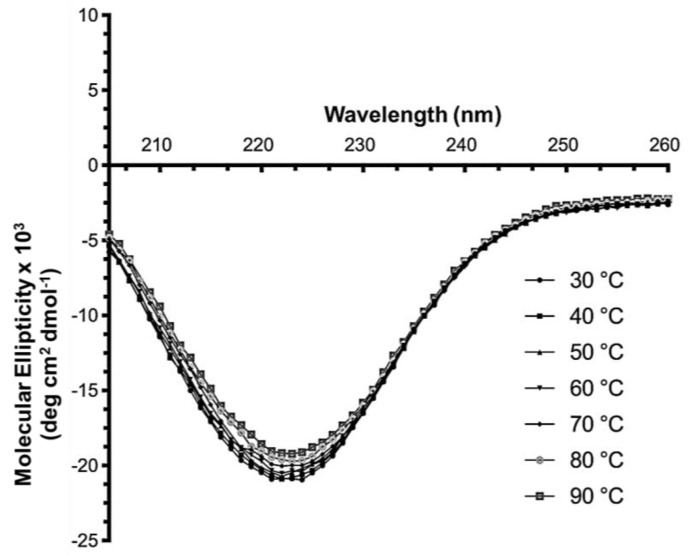
Secondary structural analysis of PPM*_Tk_* by CD spectrometry. CD spectra were recorded from 260 to 200 nm in wavelength, at a temperature range of 30–90 °C. The image was created using GraphPad Prism ver. 7.04.

The CD spectra of PPM*_Tk_* indicated the significant presence of α-helix by giving a characteristic negative peak around 222 nm. The quantitative analysis performed using CDPro [14] shows the prevalence of α-helix (49%), followed by β-sheets (20.5%), loops, and unordered structure (Figure 7). This content of secondary structure elements is comparable to those obtained from JPred4 [15] (α-helix—37% and β-sheets—23%), in silico sequence-based structure prediction using AlphaFold2 [16], and to the results obtained by X-ray crystallography. Upon temperature increase, the value of molecular ellipticity showed negligible reduction at 80–90 °C. The peak pattern remained the same in all spectra obtained from 30 °C to 90 °C, indicating that the protein maintains its conformation and the level of structural integrity.

### 2.7. Dynamic Light Scattering Analysis of PPM_Tk_

**Figure 8:**
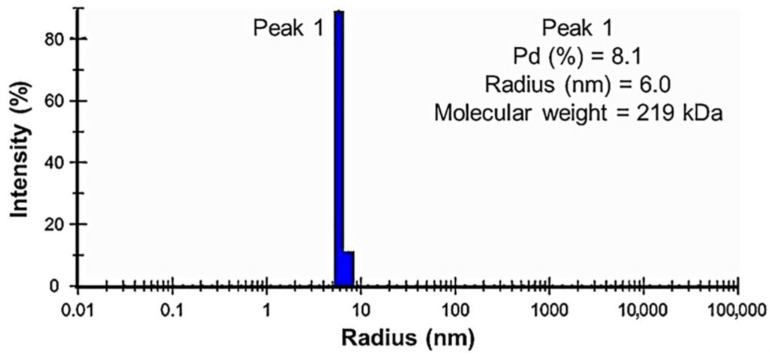
Dynamic light scattering analysis of the PPM*_Tk_* shows the particle size distribution at a concentration of 100 µM in 50 mM Tris-HCl (pH 7.0) at 22 °C.

Dynamic light scattering analysis was performed to determine the presence of any aggregates as well as the sizes and distribution of PPM*_Tk_* particles in the solution. The results showed a narrow peak for the particle size distribution (Figure 8). The polydispersity (Pd) value obtained was 8.1%, indicating a monodispersity and displaying that all the nanoparticles of PPM*_Tk_* had the same size. The hydrodynamic radius value was 6.0 nm, which corresponded to ~200 kDa when compared to the one obtained from PDBcor. These results suggested the tetrameric nature of PPM*_Tk_*.

### 2.8. Crystallization of PPM_Tk_ and Diffraction Data Analysis

**Figure 9:**
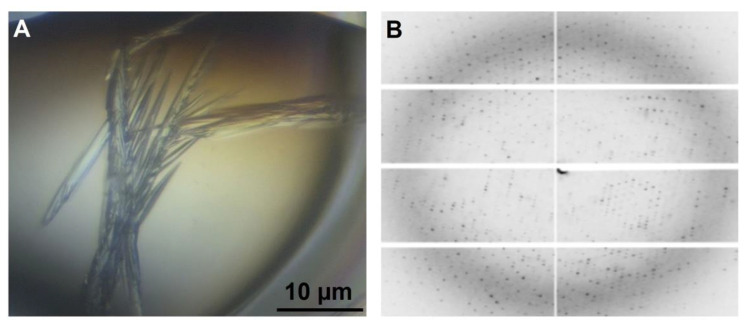
Crystals and diffraction pattern of PPM*_Tk_*. (**A**) Needle-like crystals obtained in 20% (*v*/*v*) PEG300, 100 mM Tris-HCl (pH 8.5), 5% *w*/*v* PEG8000, and 10% *v*/*v* glycerol, following sitting drop crystallization method. (**B**) The image of the diffraction pattern of PPM*_Tk_* crystals.

**Figure 10:**
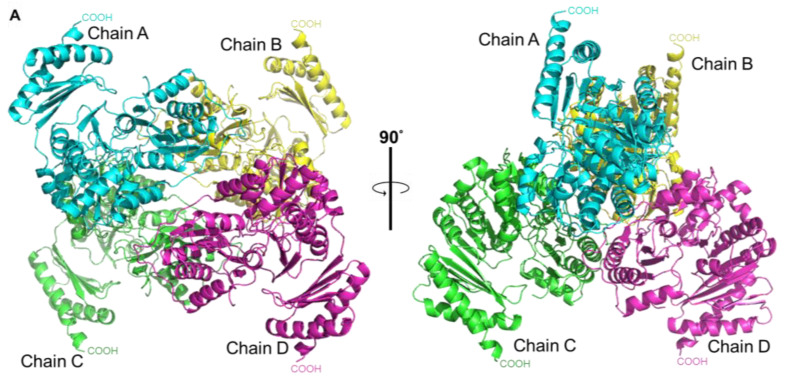

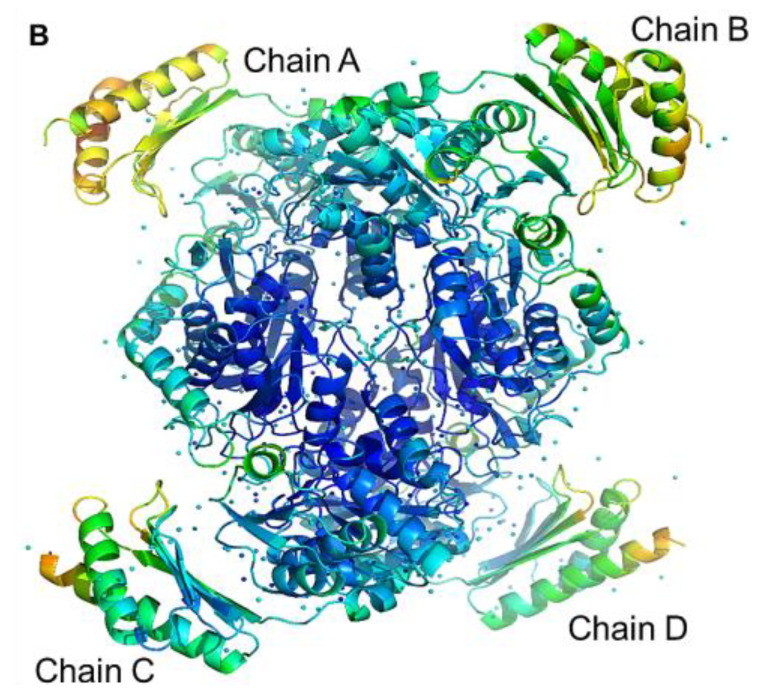
Three-dimensional structure analysis of PPM*_Tk_*. (**A**) The tetrameric structure in two different orientations. Chains A, B, C, and D are colored in light blue, yellow, green, and magenta, respectively. (**B**) Residues are colored with different shades of blue and green, according to the average values of their B-factors. The dots correspond to the water molecules.

Even though the active site of the phosphopentomutase is similar to that of phosphohexomutases, PPM*_Tk_* has been shown to be a phosphopentomutase instead of a phosphohexomutase. To understand this unique property, we crystallized PPM*_Tk_* and determined the three-dimensional structure by X-ray diffraction at a resolution of 2.39 Å (Figure 9). Crystals used for data collection were obtained in 20% (*v*/*v*) PEG300, 100 mM Tris-HCl (pH 8.5), 5% *w*/*v* PEG8000, and 10% *v*/*v* glycerol when 17 mg/mL PPM*_Tk_* was used. Crystals of PPM*_Tk_* were grown in 7 days as needles (0.1 mm long) in a monoclinic space group *P*2_1_ with four protomers in the asymmetric unit. Experimental details of data collection and refinement statistics are summarized in Table 1. The structure was solved by the molecular replacement (MR) method using a search probe model generated by the AlphaFold2 program. The Z-score after MR was 42.4 and the LLG was 20588, indicating the correctness of the structure. The structure was found to be a homotetramer (Figure 10).

### 2.9. Architecture of the Tetramer of PPM_Tk_

The relatively similar conformation of N- and C-terminal domains in all protomers of the tetramer was confirmed by doing the superimposition of Chain A to all B, C, and D chains. The RMSD values for equivalent Cα’s of the aligned residues are 0.33, 0.53, and 0.46 Å, respectively, indicating that the spatial relationship between domains is identical for all protomers in the tetramer. This shows the homotetrameric nature of PPM*_Tk_* where each subunit adopted an almost identical structure and symmetrical assembly within the tetramer.

The PDBePISA [17] (https://www.ebi.ac.uk/pdbe/pisa/ (accessed on 20 June 2024)) analysis showed that the tetramer was formed as a dimer of dimers. Upon the formation of a complete tetrameric structure, 9200 Å^2^ out of the total solvent accessible area (65140 Å^2^) was buried between the connected interfaces of all the protomers. Each of the AC and BD dimers was formed by the H-bonds between Trp^14^ (from loop 2), Thr^45^ (from loop 4), Met^49^, Ser^56^ and Thr^61^ (from α-helix 2), and Ala^132^ and Trp^134^ (from loop 8) of both the protomers. In the tetramer, chain A interacted with B and chain C interacted with D. The AB and CD assemblies were formed via hydrogen bonds and salt bridges. Some of the residues formed more than one bond involving carbon, nitrogen, and oxygen. The average H-bond length for AC (2.9 Å) and BD (2.8 Å) is comparatively shorter than those for AB (3.2 Å) and CD (3.3 Å). 

### 2.10. The Protomer of PPM_Tk_

**Figure 11:**
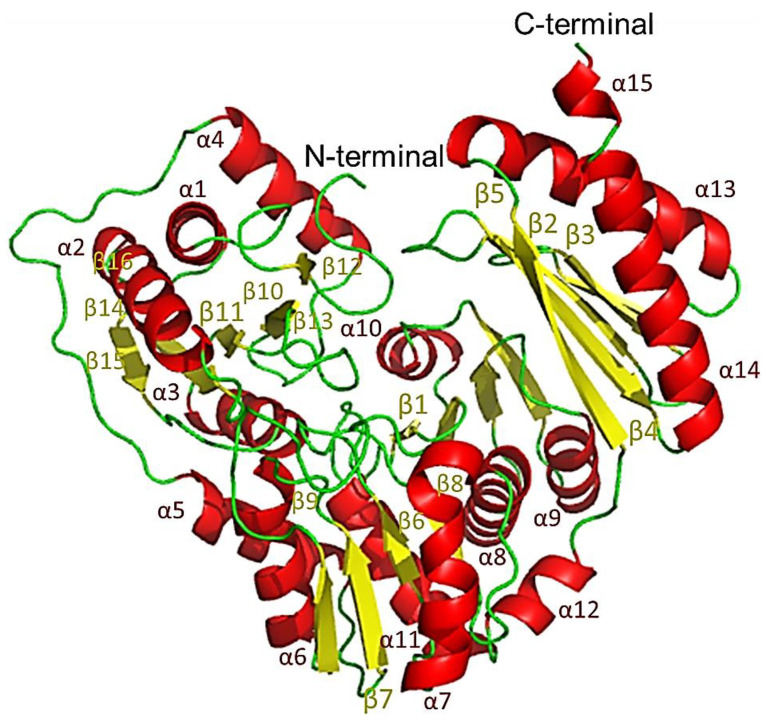
A protomer of PPM*_Tk_* showing the secondary structural elements in different colors. α-helix (red), β-sheets (yellow), and loops (green). The symbol ‘α’ shows the helices while ‘β’ represents the sheets. The numbers indicate the position in the protomer.

The protomer of PPM*_Tk_* exhibited an overall architecture of a three-layer α-β-α sandwich with a unique α/β topology and arrangement. The protomer was composed of two main domains, the N-terminal domain consisting of residues 1-350 and the C-terminal domain formed by residues 355-450. The two domains were linked via a short linker (loop 26) forming a deep cleft. Compared to the interactions between structural elements within each of the two domains, contacts between the domains were much less extensive. Each protomer contained 15 α-helices and 16 β-sheet strands (Figure 11) with the atomic displacement parameters (B-factors) widely different for different parts of the chain. Residues with high B-factors (sometimes >150 Å^2^) are more dynamic, leading to lower quality of the electron density maps in their regions. Such residues cannot be modeled as accurately as their counterparts with lower average B-factors (<30–50 Å^2^). The N-terminal domain of PPM*_Tk_* was characterized by low-to-moderate B-factors resulting from stabilizing interactions between domains from different protomers within the tetramer. C-terminal domains, however, were not significantly stabilized by interactions with other domains. As a consequence, their B-factors were high and their coordinates were less well-defined. The departure from idealized geometry and stereochemistry, reported by MolProbity [18], was almost exclusively associated with residues belonging to the C-terminal domain.

### 2.11. Structural Comparison

**Figure 12:**
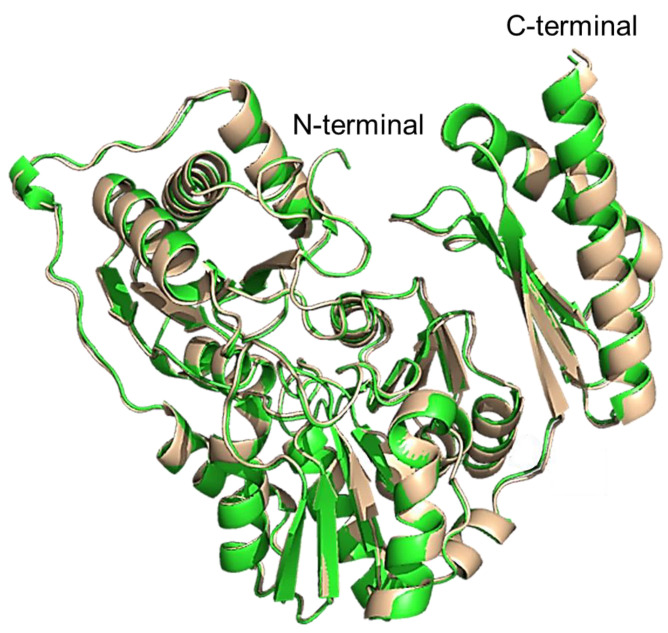
Superimposition of the protomers of experimentally determined PPM*_Tk_* (green), and AlphaFold2 predicted structure (tint). The alignment resulted in an RMSD of 0.466 Å.

The protomer of the experimentally determined structure of PPM*_Tk_* was compared with the monomeric model structure obtained using AlphaFold2. Similar to the experimentally solved structure, the AlphaFold2 model showed two main domains in the protomer. However, these domains differed in several secondary structural elements. Superimposition of the N- and C-terminal domains of the crystal structure with those of AlphaFold2 structure gave an RMSD value of 0.435 Å for 350 residues of the N-terminal domain, and 0.389 Å for the 95 residues of the C-terminal domain. Similarly, structural superposition of all the 450 residues (using GESAMT [19]; CCP4 suite, ver. 1.1.0) displayed 100% identity with an RMSD of 0.466 Å and a Q-score of 0.976, indicating a nearly perfect structural match with minimal deviation (Figure 12). However, a substantial rotation, defined by polar angles (ω = 102.53°, ϕ = −49.21°) and a rotation angle (κ) of 170.35°, was required to achieve this alignment. Following the rotation, a Cartesian shift of (x = −15.15 Å, y = −1.63 Å, z = 26.68 Å) was applied. These parameters highlight that despite perfect sequence identity, the structures require significant reorientation and translation for alignment, which may reflect conformational variability.

When the tetrameric model, predicted by AlphaFold2, was compared with that of the experimentally determined structure, an RMSD value of 34.5 Å was obtained. The AlphaFold2 modeled the backbone of the N-terminal domain of tetramer correctly but gave inaccurate modeling for the flexible C-terminal region. In addition, AlphaFold2 positioned the monomers differently in the predicted tetramer, resulting in hiding the active site residues in domain III and the C-terminal domain beneath the overall structure.

### 2.12. Comparison of PPM_Tk_ with Phosphoglucomutase

The DALI server [20] was used to compare the structure of PPM*_Tk_* and previously determined structures of homologous proteins. A phosphomannomutase/phosphoglucomutase (PGM*_Ph_*) from *Pyrococcus horikoshii* (PDB ID: 1WQA; https://doi.org/10.2210/pdb1WQA/pdb (accessed on 30 August 2024)) [21] exhibited the highest sequence identity to that of PPM*_Tk_* with a Z-score of 51.2. Both enzymes have homologous domains and motifs. An alignment of amino acid sequences of PPM*_Tk_* and PGM*_Ph_* using Clustal W (https://www.ebi.ac.uk/jdispatcher/psa/emboss_needle (accessed on 13 October 2024)) [22] demonstrated the presence of hexomutase-specific four conserved motifs in PGM*_Ph_*, while only two of them were found conserved in PPM*_Tk_*. Motif III “GEEN” was modified to “AAEP”, while motif IV “VRASGTEP” was altered to “IRPSGTEP” (Figure S5).

**Figure 13:**
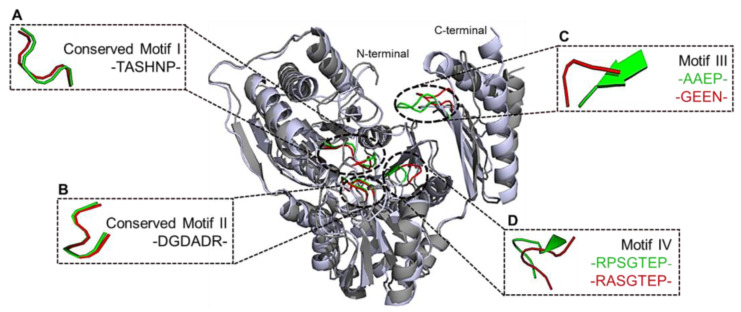
Superimposition of PPM*_Tk_* (light-grey) and PGM*_Ph_* (dark-grey) carried out using PyMol (ver. 2.3.2). Panels (**A**,**B**) show the conserved motifs. Panels (**C**,**D**) represent modified motifs (PPM*_Tk_*, green; PGM*_Ph_*, red).

A comparison of the N- and C-terminal domains of PPM*_Tk_* and PGM*_Ph_* individually using GESAMT [19] (CCP4 suite, ver. 1.1.0), yielded RMSD values of 1.465 Å for 347 N-terminal residues and 1.738 Å for 85 C-terminal residues, respectively. For all the residues, with sequence identity of 36.1%, an RMSD score of 1.85 Å and a Q-score of 0.627 showed moderate structural alignment with some conformational divergence (Figure 13). The transformation required to achieve optimal alignment involved a substantial rotation, defined by polar angles (ω = 89.58°, ϕ = 179.16°) around an axis, with a rotation angle (κ) of 95.76°. These parameters suggest that, despite moderate sequence similarity, the two structures exhibit significant orientational and positional adjustments. Moreover, the structural comparison showed that the residues Ser^93^, Asp^231^, Asp^233^ and Asp^235^ of PPM*_Tk_* and residues Ser^101^, Asp^243^, Asp^245^, and Asp^247^ of PGM*_Ph_* are involved in cofactor/metal ion binding that has been reported to involve a phosphate group (Figure S6).

### 2.13. Protein–Ligand Interaction

**Figure 14:**
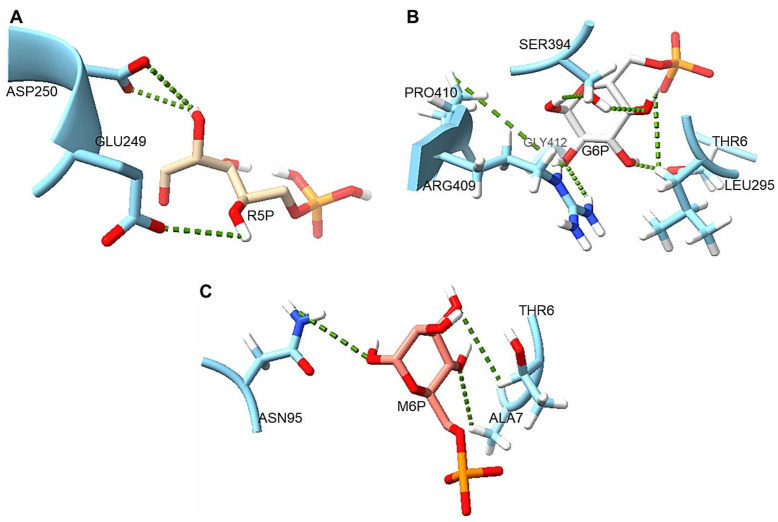
Interaction of PPM*_Tk_* with various substrate molecules: (**A**) ribose 5-phosphate (R5P), (**B**) glucose 6-phosphate (G6P), and (**C**) mannose 6-phosphate (M6P). Dotted lines show the H-bonds.

Ligand docking studies of both PPM*_Tk_* and PGM*_Ph_* with glucose 6-phosphate (G6P), mannose 6-phosphate (M6P), and ribose 5-phosphate (R5P) were performed using PoseView online (https://proteins.plus/help/poseview (accessed on 5 November 2024)) [23]. Side chains of Thr^6^, Leu^295^, Ser^394^, Arg^409^, Pro^410^, and Gly^412^ residues of PPM*_Tk_* were found to interact with G6P. Thr^6^, Ala^7^ and Asn^95^ interacted with M6P while only two residues, Glu^249^ and Asp^250^, show interaction with R5P (Figure 14). PGM*_Ph_* showed interactions between the side chains of Thr^7^, Asp^245^ and Asp^309^ with G6P. Glu^218^, Arg^248^ and Lys^374^ interacted with M6P while Thr^7^, Asp^309^, and Arg^340^ were involved in interaction with R5P. Although both proteins have been shown to have high binding affinity for these ligands, PPM*_Tk_* was not able to utilize hexoses as its substrate. Residues Arg^409^, Pro^410^ and Gly^412^ belong to the conserved motif of domain IV, reported to be involved in phosphomutase activity of hexomutases. However, none of the residues from the conserved motifs were found interacting with R5P, instead they interacted with the hexose sugar.

Our results suggest that R5P forms a smaller number of H-bonds as compared to those formed by G6P and M6P when interacting with PPM*_Tk_*. Furthermore, the average predicted lengths of hydrogen bonds (cut-off 2.5–3.5 Å) showed that G6P formed smaller H-bonds than M6P, while R5P displayed a bond length that might be suitable for the action of PPM*_Tk_*.

Although multiple sequence alignment showed the presence of conserved domains that make PPM*_Tk_* a homolog of phosphohexomutases, protein–ligand interaction studies showed that Glu^249^ and Asp^250^ of PPM*_Tk_*, which interact with the pentose, differ from their counterparts in the homologous enzymes. Moreover, these residues were not involved in binding to hexoses. This supports the observation that despite its homology to phosphohexomutases, the mechanism of activity of PPM*_Tk_* is different and that this enzyme acts solely on the pentose/ribose sugar.

## 3. Discussion

The genome sequence of hyperthermophilic archaeon *T. kodakarensis* harbors four open reading frames that are annotated as phosphomannomutase genes. All of them are members of cluster 1109 of orthologous genes (COG1109). It has been found previously that one of them, TK1777, actually encodes a phosphopentomutase (PPM*_Tk_*) [5], in contrast to all previously identified genes encoding phosphopentomutases which were members of COG1015. This study presents the biophysical characterization and crystal structure determination of this novel phosphopentomutase from *T. kodakarensis*. The crystal structure of PPM*_Tk_* contains two monomer–monomer, and one dimer–dimer interfaces stabilized by H-bonds and salt bridges. PPM*_Tk_* contains structural domains homologous to hexomutases but its catalytic activity is restricted to pentoses. Computational analysis indicated that this substrate specificity might be due to the lower activation energy barrier of pentoses, compared to hexoses, attributed to fewer interactions and longer bond lengths within the putative enzyme–substrate complex. Stronger predicted interactions with hexoses, due to more and shorter bond lengths, would require higher energy for catalysis. Although phosphoglucomutase and PPM*_Tk_* share two highly conserved active site motifs, PPM*_Tk_* differs the third substrate-binding motif [5]. The structure of PPM*_Tk_* highlights the distinct residues in the third motif that might be responsible for utilizing 5C sugars instead of 6C ones. Enzyme functioning depends upon the exact conformation of the active site and the side chains of residue involved in the catalysis. Although prediction by AlphaFold2 software is logically justified, a minor inaccuracy, particularly in flexible regions, may lead to a misinterpretation of the enzyme activity, stability and interactions with the substrates. Therefore, determining experimentally derived structures remain essential for understanding the full functional context of proteins.

Structural comparison with the homologous enzyme PGM*_Ph_* provides diverging evolutionary distance for the structural features of PPM*_Tk_* to specific functional needs. This explains how structure and function co-evolve in protein families. Besides having conserved regions as those found in PGM*_Ph_*, PPM*_Tk_* shows variations in the key secondary structural elements surrounding the active site and the overall quaternary structure, leading to changes in substrate specificity. We also performed in silico studies on the binding of R5P, G6P, and M6P to PPM*_Tk_* and PGM*_Ph_* that are in accordance with the substrate specificities reported for these enzymes. The results demonstrated that the residues involved in binding to R5P are Glu^249^ and Asp^250^ in PPM*_Tk_*. In contrast, G6P and M6P do not interact with these residues. Though both PPM*_Tk_* and PGM*_Ph_* demonstrated interaction with pentose phosphate and hexose phosphate, PPM*_Tk_* has been reported to catalyze the pentose phosphate, instead of a hexose phosphate [5]. Moreover, PGM*_Ph_* has not been experimentally validated for its activity either with pentoses or hexoses. Phosphoglucomutase (PGM*_Tk_*), another homologue of these enzymes in *T. kodakarensis*, has been experimentally studied for the high activity against hexose phosphate and a low activity against pentose phosphate [24]. PGM*_Tk_* and PGM*_Ph_* possess identical motifs I, II and III. Therefore, we postulate that PGM*_Ph_* may display a higher activity against a hexose phosphate and a lower activity against a pentose phosphate [24]. Interface analysis of the tetramer also reveals the availability of Glu^249^ and Asp^250^ for binding to the substrate. A bacterial PPM (PDB ID: 3M8Z) from *Bacillus cereus* has been reported with two mutually exclusive R5P binding positions. Position 1 comprises Thr^85^, Arg^193^ and Asp^286^, whereas position 2 consists of Thr^85^ and Ser^154^ [25]. However, PPM*_Tk_* shows no sequence and structural alignment with these residues.

Although structural homologs of PPM*_Tk_* have been identified as the phosphoglucomutases/phosphomannomutase in archaea, none of them contain identical amino acids corresponding to these two residues. The phosphopentomutase activity of PPM*_Tk_* may be attributed to the involvement of variable amino acids in domain III. These residues make a different cleft pattern in PPM*_Tk_* that allows the binding of only pentoses to the active site. This demonstrates that despite having high homology in primary protein structure and conserved regions, enzymes may function differently. To reveal the crucial residues involved in the phosphopentomutase activity of PPM*_Tk_*, computational docking results should be validated by practical elucidation of the structural dynamics of PPM*_Tk_* during catalysis. Additionally, the interaction of cofactors and hexoses with PPM*_Tk_* can be an avenue for exploring the interconnectedness of carbohydrate metabolic pathways in *T. kodakarensis*.

## 4. Materials and Methods

### 4.1. Sequence Retrieval, Physicochemical Parameters and Domain Structural Analysis

The nucleotide sequence and primary structure of the PPM*_Tk_* were obtained from GenBank and UniProt, respectively. The Expasy ProtParam tool (accessed on 1 August 2024) was used to calculate the physiochemical properties. An InterPro scan, freely available at https://www.ebi.ac.uk/interpro/ (accessed on 1 August 2024), was conducted to predict details of the domain structure and structural motifs of the protein. Protein Data Bank (PDB, https://www.rcsb.org/ (accessed on 20 June 2024)) was used to search for the reported 3D structures of phosphopentomutases, while the Biological Macromolecule Crystallization Database (BMCD, http://bmcd.ibbr.umd.edu/ (accessed on 21 June 2024)) [26] was used to search for previously reported crystallization conditions for homologous crystal structures.

### 4.2. Plasmid, Bacterial Strains, and Media

*Escherichia coli* DH5α cells (Agilent Technologies, Santa Clara, CA, USA) were used for plasmid purification, whereas *E. coli* strains BL21-CodonPlus^®^ (DE3)-RIPL and Rosetta^TM^ 2(DE3)pLysS Singles^TM^ (Agilent Technologies) were used for heterologous production of recombinant protein. Expression plasmid, pET-TK1777 [5], was used to construct gene expression plasmid.

### 4.3. Construction of the Expression Vector

The pET-TK1777 construct was modified by adding nucleotides for the His_6_-TEV (tobacco etch virus) protease cleavage site at the N-terminal of the recombinant protein. The new construct, pET-His_6_-TEV-Tk1777, was designed using the Quick-change polymerase chain reaction (PCR). Oligonucleotide forward (5′CTTTAAGAAGGAGATATACATATGGGCAGCCATCATCACCATCATCACGAGAACCTG-3′) and reverse (5′-CGTATCCCCGCTGTTCCAAAGAGCCTTGACTGGAAGTACAGGTTCTCGTGATGATGGTG-3′) primers were manually designed and sourced from Integrated DNA Technologies, USA. The PCR product of the first reaction was purified (QIAquick PCR Purification Kit; Cat. No. 28104) and used as a primer for the second reaction. After PCR amplification, the reaction mixture was treated with restriction enzyme *Dpn*I to digest the methylated template DNA and to select the newly synthesized DNA. The recombinant construct, pET-His_6_-TEV-Tk1777, was used to transform *E. coli* DH5α cells. Cloning of the newly synthesized gene was confirmed by restriction digestion and DNA sequencing.

### 4.4. Optimization of the Expression of Tk1777 Gene in E. coli

Heterologous gene expression was optimized in *E. coli* BL21-CodonPlus^®^ (DE3)-RIPL and Rosetta^TM^ 2(DE3)pLysS Singles^TM^ cells. Chemically prepared both types of competent cells were transformed with 200 ng of pET-His_6_-TEV-Tk1777 and spread on LB-agar plates supplemented with ampicillin and chloramphenicol. A single colony of the transformed cells was inoculated in LB broth containing ampicillin and chloramphenicol and incubated at 37 °C. Heterologous gene expression was induced with a 0.1 mM (final concentration) of Isopropyl ß-D-1-thiogalactopyranoside (IPTG), when an OD_600_ of 0.6 was achieved. The culture was incubated for an additional 4 h under the same conditions.

### 4.5. Cell Lysis and Partial Protein Purification

Cells were harvested by centrifugation at 5000× *g* and 4 °C for 20 min. The cell pellet was washed and resuspended in 50 mM Tris-HCl (pH 8.0) containing 500 mM NaCl. The cells were lysed in a cold room using a French press at 950–1000 psi. The soluble and insoluble fractions were separated by centrifugation at 30,000× *g* for 20 min at 10 °C (Beckman Avanti™ JXN-30 High-Performance Centrifuge, Rotor JA-25.50; Beckman Coulter, Brea, CA, USA). The soluble fraction, after centrifugation, was incubated at 85 °C for 30 min and subsequently placed on ice for 30 min. The coagulated heat-labile proteins of *E. coli* origin were removed by another centrifugation at 30,000× *g* and 10 °C for 30 min.

### 4.6. Purification of Recombinant PPM_Tk_

Partially purified PPM*_Tk_* in the soluble fraction, after heat treatment, was filtered through a 0.45 µm filter device and loaded onto a Ni-NTA affinity chromatography column (HisTrap^TM^ HP; Product No. 17524802) equilibrated with Buffer A (50 mM Tris-HCl pH 8.0, containing 500 mM NaCl). After washing the column with 20 column volumes of Buffer A, PPM*_Tk_* was eluted isocratically with Buffer B (500 mM imidazole in 50 mM Tris-HCl pH 8.0, containing 500 mM NaCl). Fractions comprising a significant amount of PPM*_Tk_* were pooled and treated with TEV protease (50 µg/mL). The sample was dialyzed against 50 mM Tris-HCl pH 8.0, containing 200 mM NaCl. The dialyzed sample was reapplied to the Ni-NTA column to remove the uncleaved fraction. The untagged PPM*_Tk_* molecules were separated in the flow-through fraction, whereas the tagged protein remained bound to the column and was eluted later. The cleaved PPM*_Tk_* was concentrated using Amicon^®^ Ultra-15 Centrifugal Filter Units (Merck Millipore, Burlington, MA, USA).

### 4.7. Estimation of Molecular Weight by Gel Filtration Chromatography

To assess the molecular weight and subunit number, the concentrated sample was applied onto a HiLoad^TM^ 16/60 Superdex^TM^ 200 Prep Grade column (Cytiva, Marlborough, MA, USA) equilibrated with 50 mM HEPES pH 7.0, containing 200 mM NaCl. Purified PPM*_Tk_* was analyzed by denaturing as well as non-denaturing PAGE.

### 4.8. Determination of Molecular Weight Using ESI-MS

To verify the purity and molecular weight, PPM*_Tk_* was analyzed by LC-MS using an Agilent Technologies (Santa Clara, CA, USA) 6130 Single Quadrupole LC/MS System. Sample preparation involved mixing 45 µL of MS buffer (20% isopropanol containing 10% acetic acid and water) with 5 µL of 2 mg/mL protein sample. The LC part of the experiment was performed using an injection volume of 10 µL on an Agilent Technologies Poroshell StableBond 300 C3 column with a mobile phase of 95% H_2_O: 5% Acetic acid, and 5% H_2_O: 95% Acetonitrile, at 40 °C. The MS analysis employed ESI ionization at the capillary voltage of 3.5 kV, with N_2_ drying gas at 12 L/min and 350 °C, and nebulizing gas N_2_ at 50 psi.

### 4.9. Mass Photometric Analysis of PPM_Tk_

For mass photometric analysis, PPM*_Tk_* was diluted to 75 nM, 50 nM, and 31.25 nM in 50 mM HEPES, pH 7.0 containing 200 mM NaCl. A standard curve was drawn using freshly prepared working solutions of Thyroglobulin (Tg: 330 and 660 kDa), and β-amylase (BAM: 56, 112, and 224 kDa). Mass photometry [27] scan was run employing a OneMP mass photometer using the program Acquire MP ver. 2024 R1 (Refeyn Ltd., Oxford, UK), and the data generated were analyzed by DiscoverMP software ver. 2024 R1 (Refeyn Ltd., Oxford, UK). Raw contrast values were converted to molecular mass using a standard mass calibration with binding events grouped in a 5 kDa bin width.

### 4.10. Differential Scanning Fluorimetry Analysis

Differential scanning fluorimetry was performed to assess the thermal stability of PPM*_Tk_* under various pH conditions. Buffers used were: Bicine (pH 9.0), Tris-HCl (pH 9.0 and 8.0), and HEPES (pH 7.0). In DSF, the melting temperature (T_m_) of a protein is measured as the ratio of tryptophan fluorescence at 330 nm and 350 nm after UV excitation at 280 nm [28]. PPM*_Tk_* (2 mg/mL) was loaded on Prometheus NT 48 Capillaries Fluorimeter (NanoTemper Technologies, Munich, Germany). Following UV excitation at 280 nm, fluorescence was scanned from 20 to 95 °C at a rate of 1 °C/min. The collected data measurements were analyzed using PR.ThermControl software ver. 2.1.6 (NanoTemper Technologies, Munich, Germany).

### 4.11. Secondary Structure Analysis by CD Spectrometry

For CD spectrometry, a protein sample (0.3 mg/mL) was prepared in 10 mM Tris-HCl (pH 8.0), and measurements were recorded in the far-UV region (260–200 nm) at 30–90 °C range of temperature using Jasco (Easton, MD, USA) Circular Dichroism Spectrophotometer. The final spectrum was obtained in a single scan and the CD spectrum of the blank was subtracted from the sample spectrum. The mean residual ellipticity was calculated using the protein’s mean residual weight. Results were analyzed using CDPro [14], a desktop software for protein secondary structure analyses from CD spectroscopic data (https://sites.google.com/view/sreerama (accessed on 13 August 2024)), with the following specific parameters set: file format as Free (with preview), input unit (millidegrees/theta), initial wavelength (260 nm), final wavelength (200 nm), wavelength step (1 mm), analysis program Spectra Manager Version 2.15.03 (Build 1), reference dataset 4 and Output unit in delta epsilon.

### 4.12. Dynamic Light Scattering Analysis

Dynamic light scattering analysis was performed to determine the homogeneity level and size [29]. PPM*_Tk_* was stored at −80 °C for 1 week and then thawed slowly on ice just before the experiment. A 100 µM protein sample was prepared in 50 mM HEPES buffer (pH 7.0) containing 200 mM NaCl. The sample and blank were loaded on the standard plate Bio-one 1536 well plate (Greiner, Monroe, NC, USA) and air bubbles were removed by centrifugation. Measurements were taken using a DynaPro Plate Reader (Wyatt Technology Corporation, Goleta, CA, USA) at 22 °C with an acquisition time of 5 sec and default parameters. Data analysis was performed using DYNAMICS software ver. 8.2.0.297 (Wyatt Technology Corporation).

### 4.13. Crystallization, Diffraction Data Collection, and Data Processing

PPM*_Tk_*, at a concentration of 17 mg/mL in 50 mM HEPES (pH 7.0) containing 200 mM NaCl, was crystallized by vapor diffusion at the Structural Biology Research Centre, High Energy Accelerator Research Organization, Japan. The experiments were set up using a crystallization robot from SPT LabTech, Melbourne, UK (Mosquito Xtal) with 0.2 µL protein drop and 0.2 µL of reservoir solution in a 96-well crystallization plate at 20 °C. Hits were identified by imaging the plates using Rock Imager 2 (Formulatrix, Dubai, UAE). The initial hits were subjected to quality assessment by placing them in an X-ray beam and evaluating the resulting diffraction. Fine crystals were used for the final X-ray diffraction data collection at the Photon Factory, High Energy Accelerator Research Organization, KEK, Japan. Data were processed using the program XDS [30]. The structure was solved by molecular replacement (MR) with the program Phaser [31] that performs rigid body refinement (CCP4 suite, ver. 1.1.0 [32]) and the PPM*_Tk_* model generated by the program AlphaFold2 [16] as a search probe. The model can also be downloaded from the UniProt database, entry Q6I7B6 (https://alphafold.ebi.ac.uk/entry/Q6I7B6 (accessed on 10 December 2023)).

The MR search was set for identifying four protomers, the composition assumed based on results of biophysical experiments (i.e., mass photometry or dynamic light scattering), described in this manuscript as well as calculation of the most probable solvent content. Subsequently, the structure was refined with the program REFMAC5 (ver. 5.8.0258) [33] at a resolution range of 40–2.39 Å and extended over residues 3 through 447 in each of the four protomers. All rounds of refinement were interspersed with manual corrections to the structure using program Coot (ver. 0.8.9.2) [34]. Based on the visible peaks in the 2mF_o_-DF_c_ and mF_o_-DF_c_ electron density maps, 420 water molecules were added to the structure. Furthermore, two fragments of polyethylene glycol (PEG300) and one molecule of glycerol were modeled based on the shape and extent of the electron density peaks. The atomic displacement parameters (B-factors) were refined according to the isotropic model for individual atoms.

### 4.14. In Silico Analysis of PPM_Tk_

To predict the binding affinity and orientation of ligands, PPM*_Tk_* was subjected to protein–ligand docking using AutoDock Vina [35] and Webina web server (https://durrantlab.pitt.edu/webina/ (accessed on 15 August 2024)) [36]. Considering the specification of AutoDock Vina, the protein receptor was prepared by removing water molecules, and adding the polar hydrogens. The 3D coordinates for the ligand were generated after assigning atom types and charges using Open Babel [37]. A box center was set at 13 × 90 × 70 Å, followed by a grid box with dimensions 30 × 30 × 30 Å, and grid spacing 1.0 Å defined around the receptor’s binding pocket. The exhaustiveness was kept at 8 by default. Nine binding poses were obtained as an output, each with a calculated binding affinity in kcal/mol. After running the docking process, the resulting binding poses and affinities were analyzed to determine the most favorable interaction between the ligand and receptor. The results were analyzed using ChimeraX (ver. 1.9) [38]. Homology analysis of PPM*_Tk_* was performed using the DALI server (http://ekhidna2.biocenter.helsinki.fi/dali/ (accessed on 25 July 2024)) [20] and the structural homologs were further used for comparison.

### 4.15. Structure Deposition

Atomic coordinates and structure factors have been deposited in the Protein Data Bank under PDB entry 9IX8.

## Figures and Tables

**Table 1:** X-ray crystallographic data collection and refinement statistics for PPM*_Tk_*.

Data Collection	
Source	Photon Factory beamline BL-1A
Detector	Eiger X 4M
Wavelength (Å)	1.90
Data collection temperature (K)	100
Resolution range (Å)	50.0–2.39 (2.53–2.39) *
Space group	*P*2_1_
*a*, *b*, *c* (Å); β (°)	79.77, 97.58, 128.15; 94.0
Total reflections	539,005 (86105)
Unique reflections	77,057 (12,137)
Multiplicity	7.0 (7.9)
Completeness (%)	98.9 (97.0)
Mean *I*/σ(*I*)	10.5 (1.6)
*R*_merge_	0.135 (1.12)
CC_1/2_	0.998 (0.70)
**Refinement**	
Reflections used in refinement	74,699 (5367)
Reflections used for *R*_free_	2310 (166)
*R*_work_	0.201 (0.373)
*R*_free_	0.274 (0.391)
Matthews coefficient (Å)	2.46
Solvent content (%)	49.97
**No. of atoms**	
Protein	13,829
Ligands	48
Water	420
No. of protein residues	1780
**R.M.S. deviations**	
Bond lengths (Å)	0.010
Bond angles (°)	1.72
**Ramachandran plot**	
Favored (%)	93.4
Outliers (%)	1.2
Rotamer outliers (%)	6.1
Clashscore	6.9 (98th percentile)
***B* factors (Å^2^)**	
Average	50.5

* Values in parentheses are for the highest resolution shell.

## Data Availability

The data presented in this study are openly available in Protein Data Bank (https://www.rcsb.org/structure/unreleased/9IX8 (accessed on 26 November 2024)).

## Supplementary Materials

The following supporting information can be downloaded at: https://www.mdpi.com/article/10.3390/ijms252312893/s1.
