# Supplementary material for: Biophysical Characterization of a Novel Phosphopentomutase from the Hyperthermophilic Archaeon Thermococcus kodakarensis
Source: Int J Mol Sci. 2024 Nov 30;25(23):12893. doi: 10.3390/ijms252312893 (PMC11641776; doi:10.3390/ijms252312893)
Supplement: Supplementary file 1 [file ijms-25-12893-s001.zip › ijms-3288033-supplementary.pdf]

Supplementary data for the manuscript entitled: "Biophysical characterization of a novel phosphopentomutase from the hyperthermophilic archaeon *Thermococcus kodakarensis*"

1. *Construction of the expression vector, pET-His$_6$-TEV-Tk1777, using Quick-change PCR*

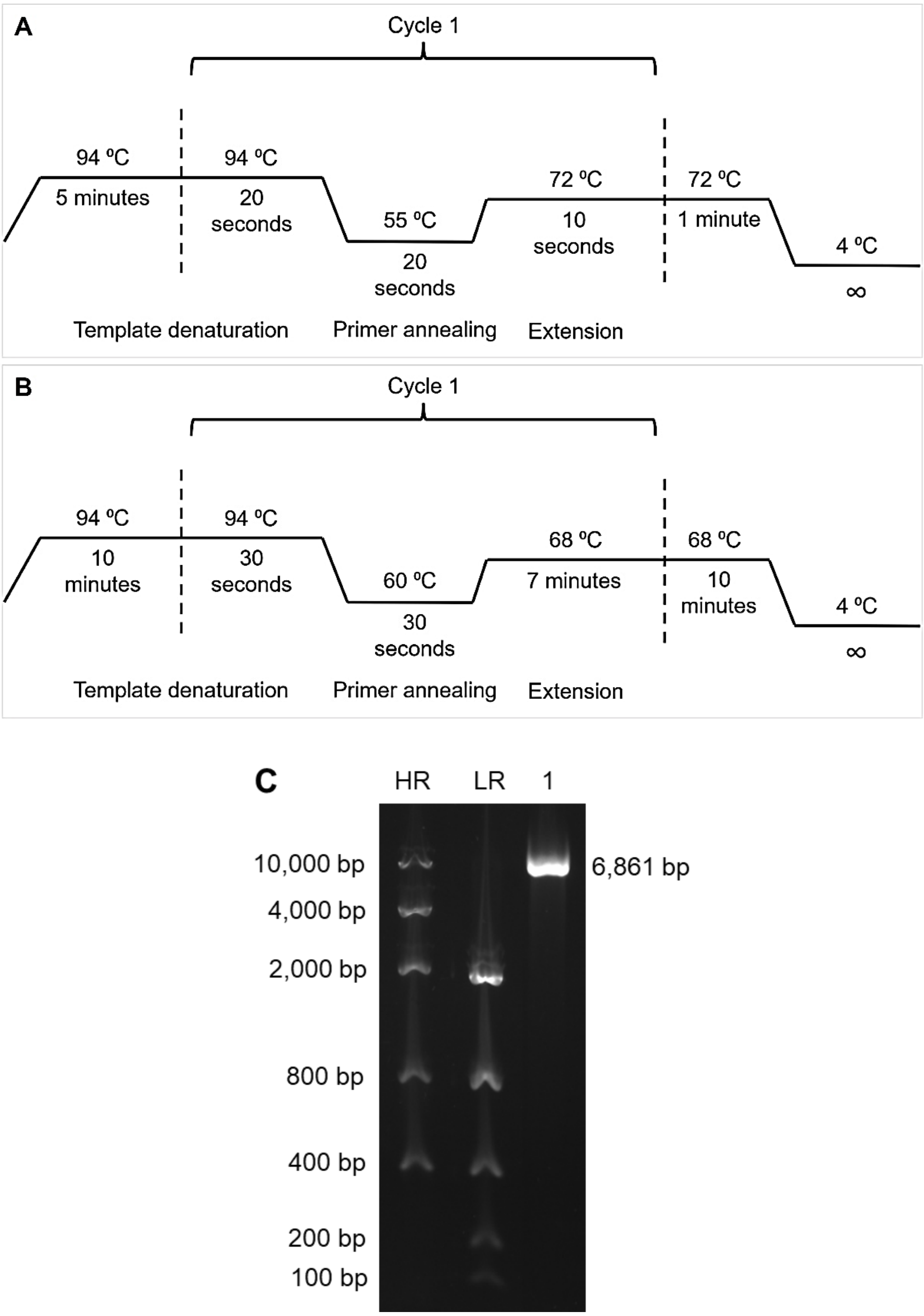

Figure S1. Pictorial representation of Quick-change PCR steps for constructing the expression vector, pET-His$_6$-TEV-Tk1777. (A) step 1: primer annealing and extension, (B) step 2: construct extension, (C) agarose gel showing: HR: Invitrogen E-Gel™ 96 High Range DNA Ladder (Product No. MAN0001082); LR: Invitrogen E-Gel™ Low Range Quantitative DNA Ladder (Product No. MAN0001085); Lane 1: purified PCR product (6,861 bp).

2. *Restriction digestion analysis of the expression vector, pET-His$_6$-TEV-Tk1777*

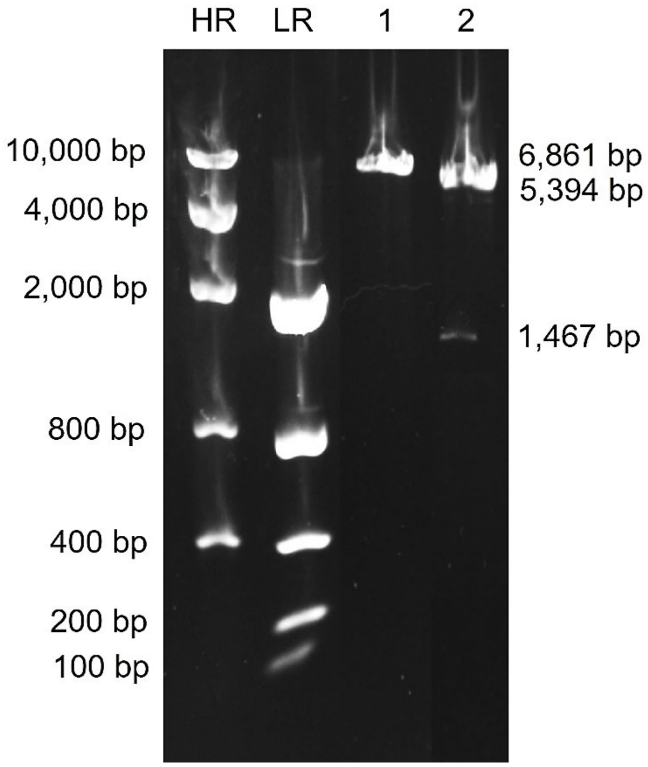

Figure S2. Agarose gel (Invitrogen E-Gel™ EX Agarose Gels, Cat. No. G401001) for restriction digestion analysis of the construct pET-His$_6$-TEV-Tk1777) with *Eco*R1 and *Nde*I. HR: Invitrogen E-Gel™ 96 High Range DNA Ladder (Product No. MAN0001082); LR: Invitrogen E-Gel™ Low Range Quantitative DNA Ladder (Product No. MAN0001085); Lane 1 shows the undigested construct (6,861 bp); and Lane 2 shows the bands of size 1,467 bp, and 5,394 bp, obtained after restriction digestion.

3. *Sequencing electropherogram for His$_6$-TEV-Tk1777*

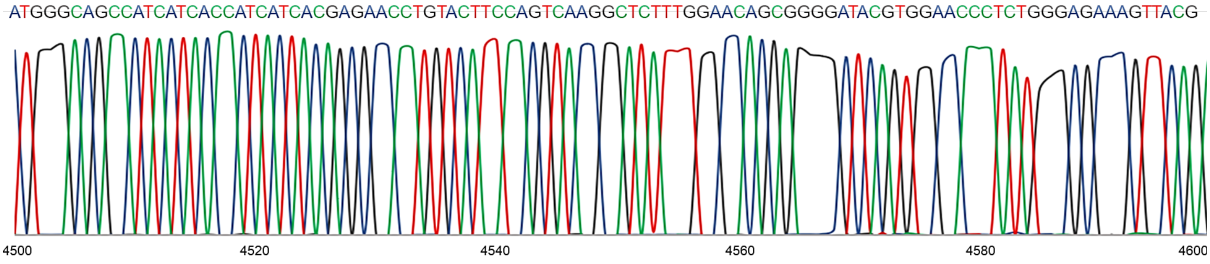

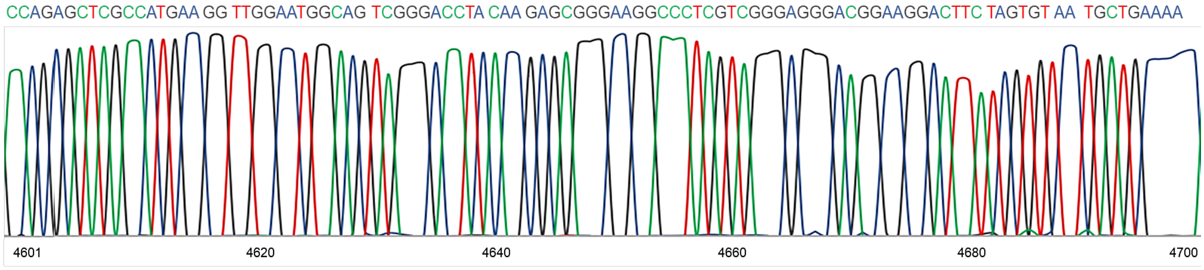

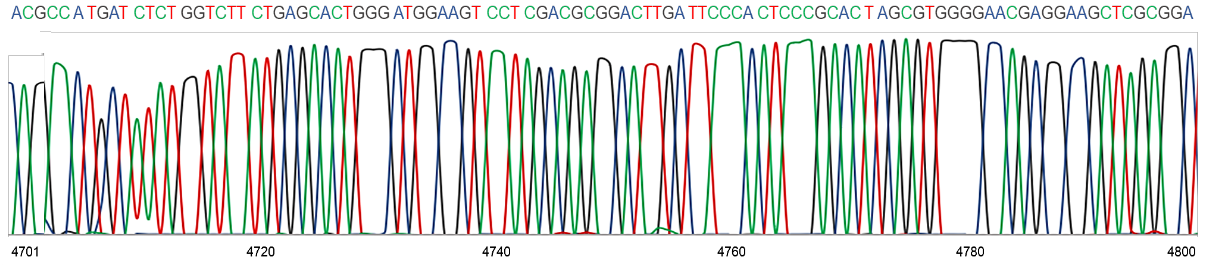

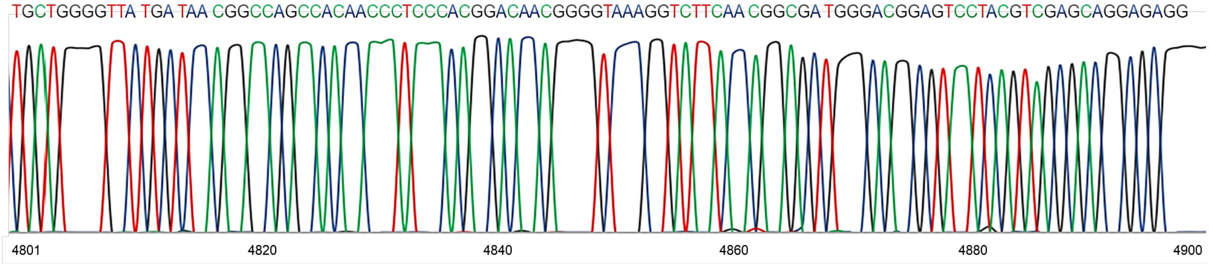

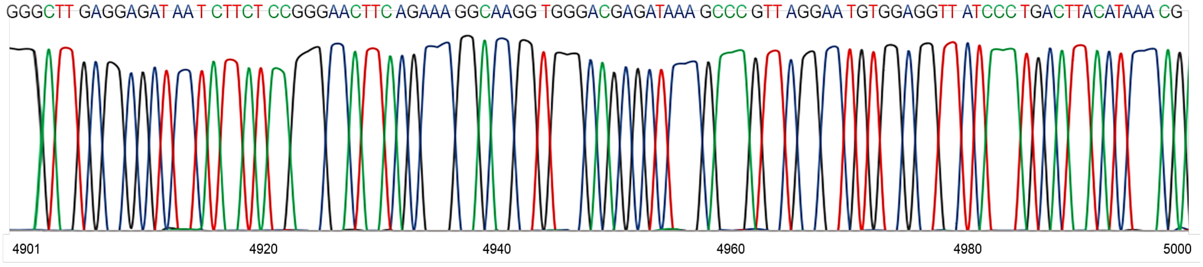

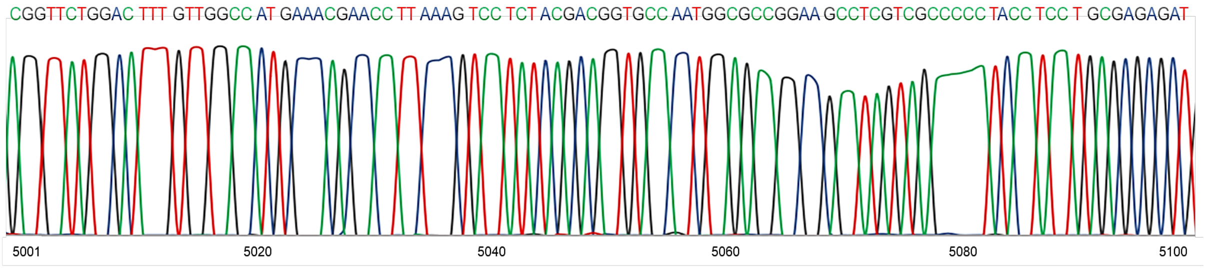
CGGTTCTGGAC TTT GTTGGCC AT GAAACGAACC TT AAAG TCC TCT ACGACGGTGCC AATGGCGCCGGAA GCCTCGTCGCCCCC TACC TCC T GCGAGAGAT
5001
5020
5040
5060
5080
5100

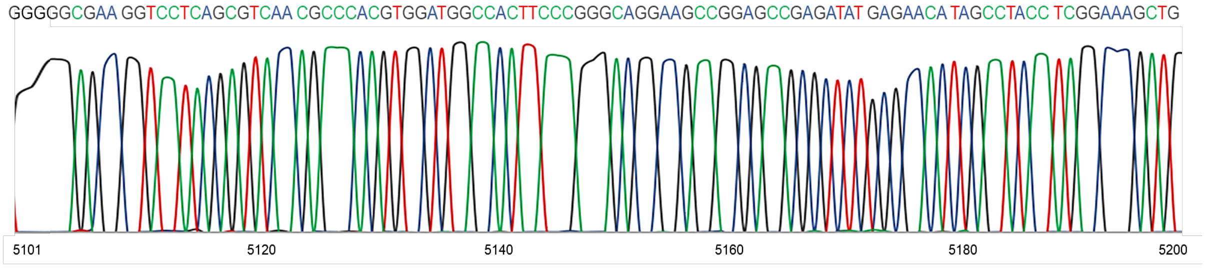
GGGGGCGAA GGTCCTCAGCGTCAA CGCCCACGTGGATGGCCACTTCCCGGGCAGGAAGCCGGAGCCGAGATAT GAGAACA TAGCCTACC TCGGAAAGCTG
5101
5120
5140
5160
5180
5200

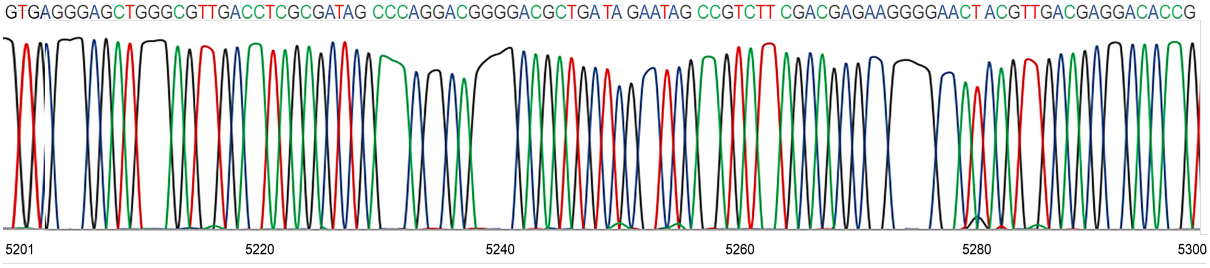
GTGAGGGAGCTGGGCGTTGACCTCGCGATAG CCCAGGACGGGGACGCTGA TA GAATAG CCGTCTT CGACGAGAAGGGGAACT ACGTTGACGAGGACACCG
5201
5220
5240
5260
5280
5300

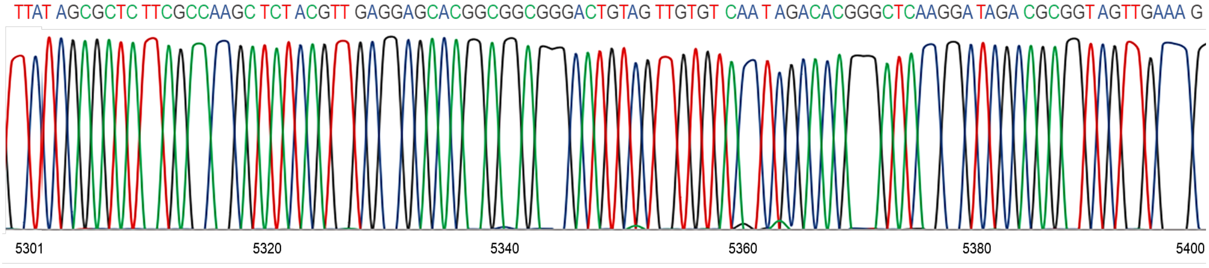
TTAT AGCGCTC TTCGCCAAGC TCT ACGTT GAGGAGCACGGCGGCGGGACTGTAG TTGTGT CAA T AGACACGGGCTCAAGGA TAGA CGCGGT AGTTGAAA G
5301
5320
5340
5360
5380
5400

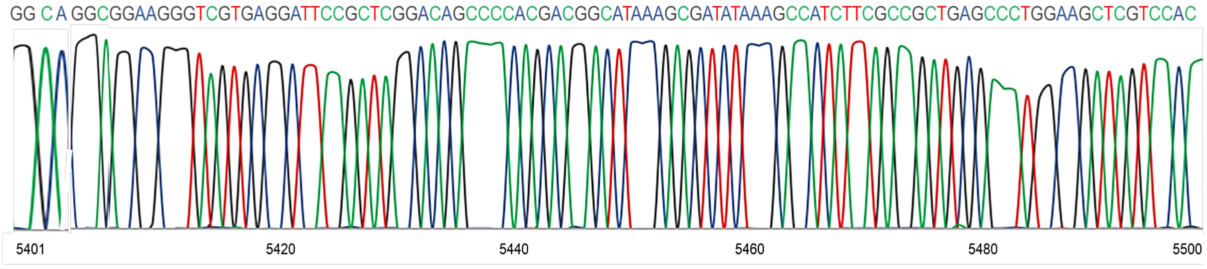
GG C A GGCGGAAGGGTCGTGAGGATTCCGCTCGGACAGCCCCACGACGGCATAAAGCGATATAAAGCCATCTTCGCCGCTGAGCCCTGGAAGCTCGTCCAC
5401
5420
5440
5460
5480
5500

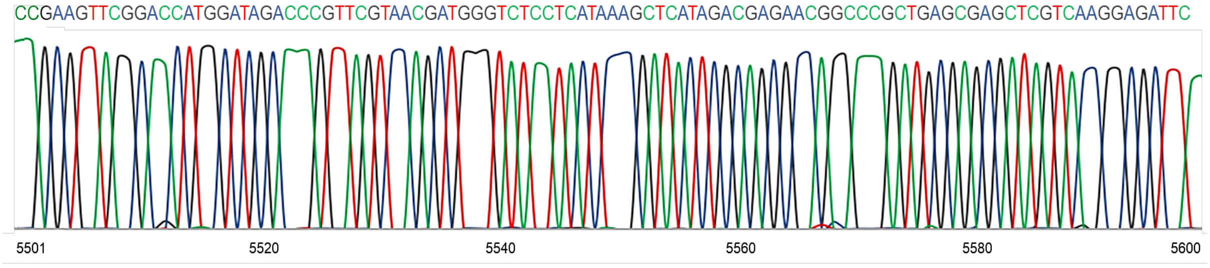
CCGAAGTTCGGACCATGGATAGACCCGTTCGTAACGATGGGTCTCCTCATAAAGCTCATAGACGAGAACGGCCCGCTGAGCGAGCTCGTCAAGGAGATTC
5501
5520
5540
5560
5580
5600

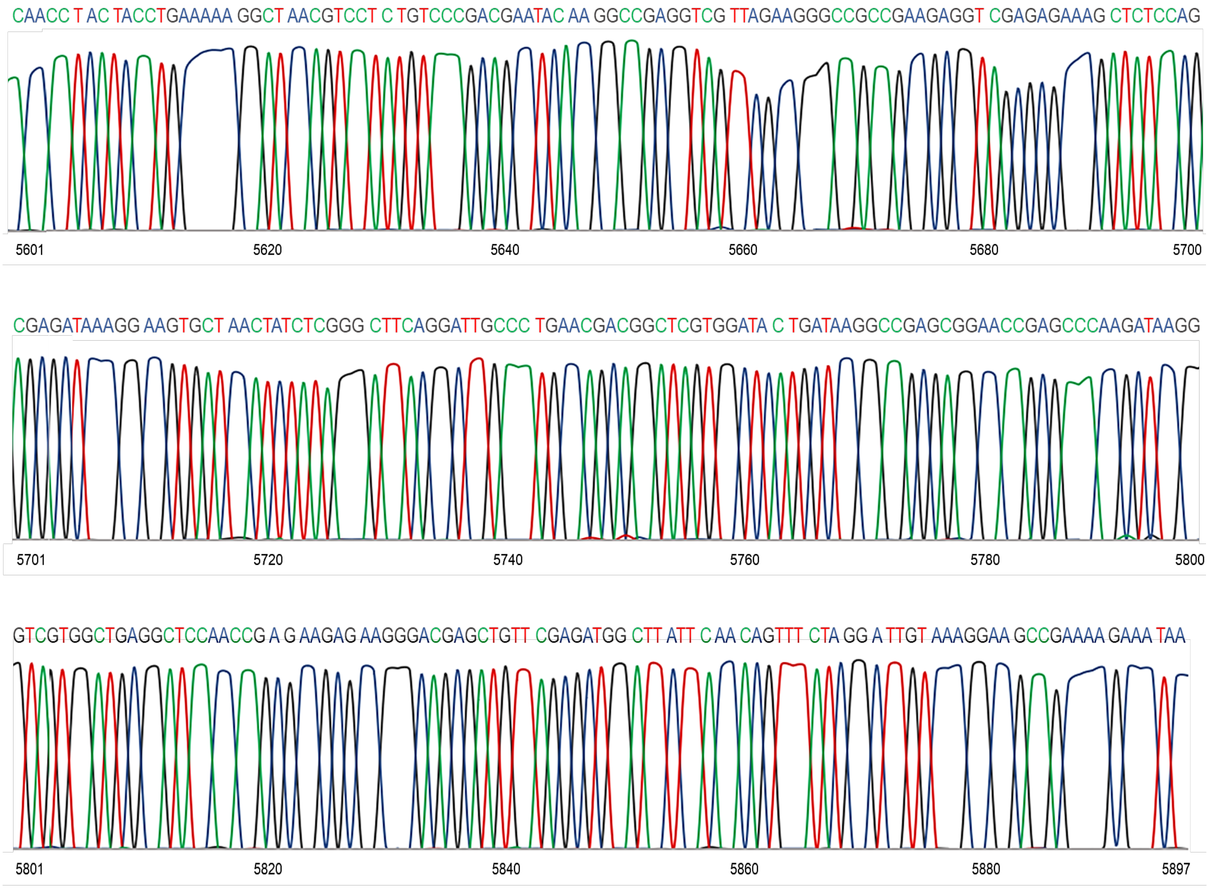

Figure S3. The computer-generated electropherogram shows the nucleotide sequencing for His$_6$-TEV-Tk1777. The expression construct was subjected to Sanger sequencing to confirm the product of Quick-change PCR.

4. SDS-PAGE analysis of the expression of His6-TEV-Tk1777 in E. coli strains

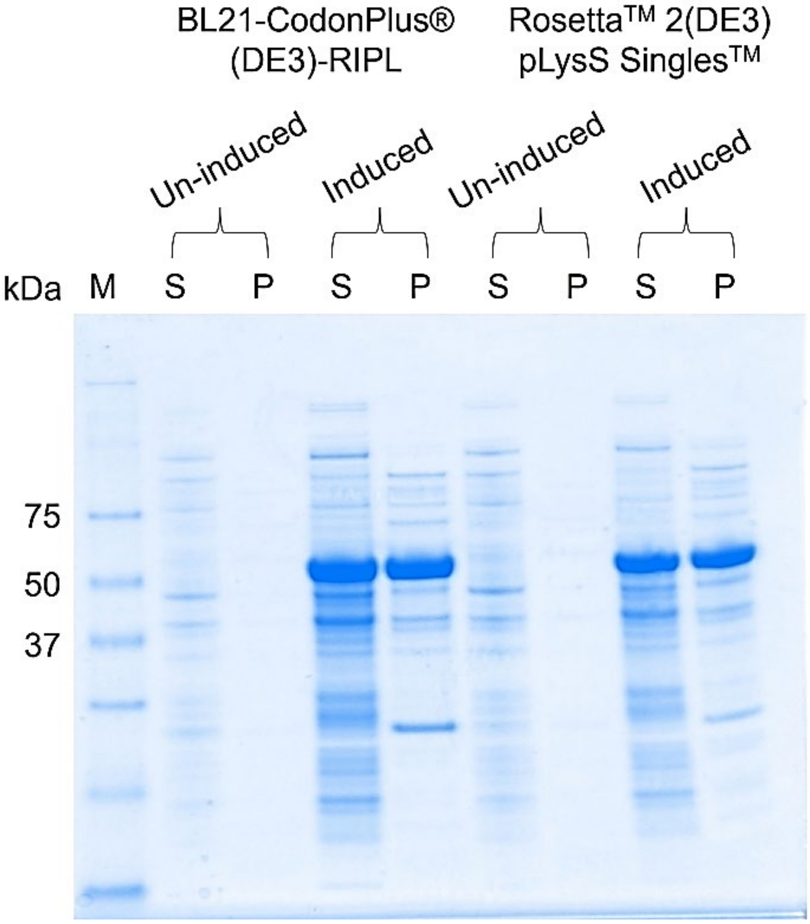

Figure S4. SDS-PAGE analysis of the expression of His6-TEV-Tk1777 in *E. coli* strains; BL21-CodonPlus®(DE3)-RIPL, and Rosetta™ 2(DE3) pLysS Singles™. Protein expression was carried out at 18 °C, using 1 mM IPTG as an inducer. Lane S shows the soluble fractions while Lane P shows inclusion bodies. Lane M is the protein marker (BioRad_Precision Plus Protein™ Unstained Protein Standards, Cat. #1610363).

5. *Sequence alignment of PPM$_{Tk}$ and PGM$_{Ph}$*

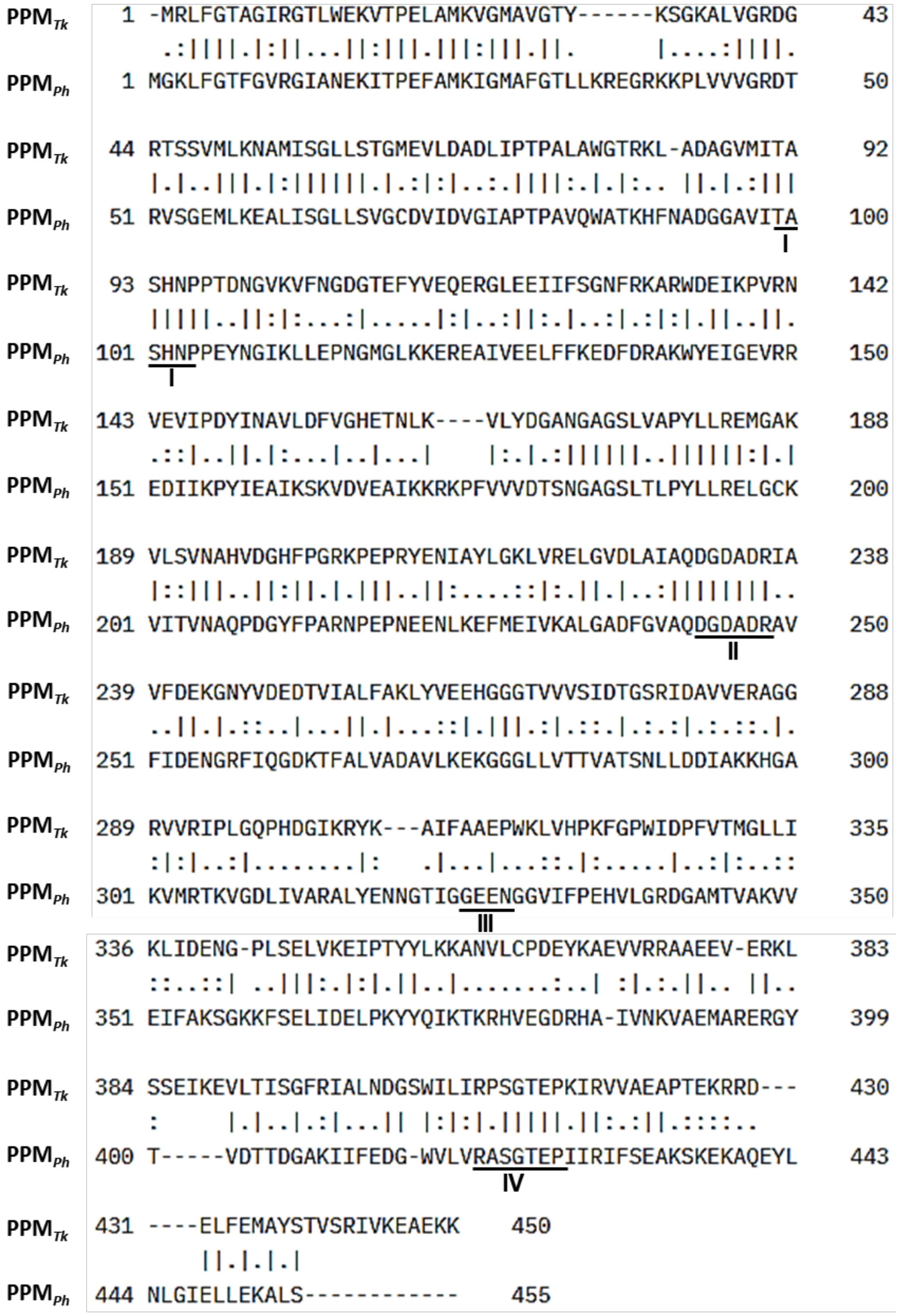

Figure S5. Pair-wise sequence alignment (PSA) of PPM$_{Tk}$ and PGM$_{Ph}$ done using Clustal W shows presence of four conserved motifs in PGM$_{Ph}$. For PPM$_{Tk}$, motif I and II are present while the motifs III and IV have different amino acid residues. Symbol 'I' (bar) shows the identical residues; a ':' (colon) highlights residues with strongly similar properties; a '.' (period) indicates residues with weekly similar properties; and a '-' (hyphen) shows the gap.

6. *Cofactor binding site of PPM_{Tk} and PGM_{Ph}*

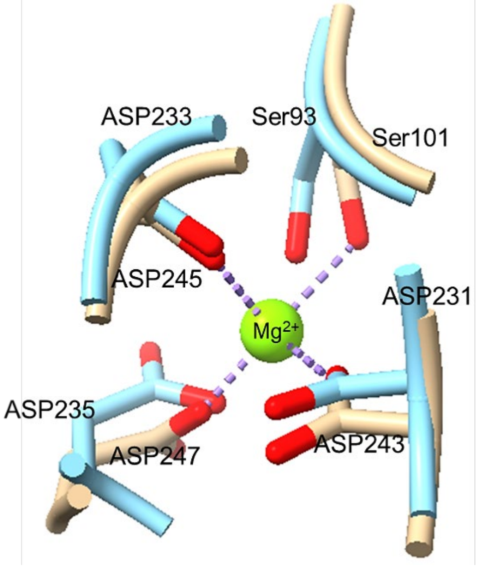

Figure S6. Structural alignment of PPM_{Tk} and PGM_{Ph} done using ChimeraX (ver. 1.9) showed the well-aligned cofactor binding site. Magnesium (MG^{2+}) helps stabilize the overall protein structure and supports binding of the substrate to the active site cleft. Cofactor binding residues of PGM_{Ph} (in tint color): Ser^{101}, Asp^{243}, Asp^{245}, and Asp^{247}; of PPM_{Tk} (in light-blue color): Ser^{93}, Asp^{231}, Asp^{233}, and Asp^{235}.
